# Adverse experiences of women with undiagnosed ADHD and the invaluable role of diagnosis

**DOI:** 10.1038/s41598-025-04782-y

**Published:** 2025-07-01

**Authors:** Eve Holden, Helena Kobayashi-Wood

**Affiliations:** 1 School of Psychology and Neuroscience, University of St Andrews, St Andrews, UK; 2 Department of Psychology, Durham University, Durham, UK

**Keywords:** ADHD, Attention deficit hyperactivity disorder, Neurodivergence, Late-diagnosis, Lived experience, Neurodivergent women, Psychology, ADHD, Diagnosis

## Abstract

Girls and women are disproportionately affected by delayed diagnoses of attention-deficit/hyperactivity disorder (ADHD), but research on the impact of this is limited. Our study aimed to centre lived experiences of women with late-diagnosed ADHD to increase understanding of the impact of such delays. We employed a mixed methods survey to investigate the perspectives of 28 women with late-diagnosed ADHD. Results starkly demonstrate the criticism and lack of support participants faced from society and medical professionals, illustrating the negative consequences of delayed ADHD diagnosis on quality of life and mental health. Participants commonly reported internalising criticism and described disconcertingly low self-esteem; citing guilt, shame, and negative self-perception due to delayed diagnoses. Participants found diagnosis revelatory, their lives finally making sense; citing healing, improved self-esteem, and life feeling more worth living. The adversities faced from delayed diagnoses were described from early childhood, through adolescence, and adulthood. Participants reflected on ‘what could have been’, and described grieving the lives they could have led if diagnosed earlier. The negative impacts of missed ADHD diagnosis are broad and span life stages. With potential implications for public health policy, this work highlights the importance of increasing girls’ and women’s access to ADHD diagnoses to address avoidable detrimental hardship.

## Introduction

There have been widespread misconceptions about Attention Deficit/Hyperactivity Disorder (ADHD) as being the ‘hyperactive boy’ disorder; and people who present differently to the misconceived norm, most commonly girls, are more likely to go undiagnosed or are diagnosed as adults^1,2^. The impact of misconceptions and biases, both past and present, on women with ADHD are significant and widespread. Delayed diagnoses can not only have negative effects on academic achievement and career success but can also negatively impact wellbeing and self-esteem^3–6^. However, perspectives from women on the impact of going for years undiagnosed are not well documented. It is still unclear how delayed diagnoses impact women of varied ages, how they perceive living undiagnosed impacted different stages of their lives, and what the diagnosis meant to them. It is imperative that these voices are represented and taken into consideration when working towards increasing support post-diagnosis, and in demonstrating the importance of increasing access to diagnosis. In this piece of work we explore women’s first-hand perspectives on receiving delayed ADHD diagnosis.

ADHD is most commonly characterised by the presentation of symptoms of inattention and hyperactivity^7^. However, some models of ADHD outline how symptoms can better be characterised from a more top-down approach by describing deficiencies in executive function^8–10^. For example, executive function deficiencies can present as difficulties with self-regulation, which can manifest in attention regulation deficiencies (e.g. being easily distracted, or engaging in ‘hyperfocus’), difficulties regulating emotions, and difficulties inhibiting impulsive behaviours^8^. Symptoms of self-regulation deficiencies can present outwardly as external hyperactivity but can also present internally, for example, via racing thoughts or psychological restlessness^11–14^.

Since ADHD affects many areas of cognitive functioning, it is unsurprising that it has severe impacts on people’s experiences of life: including abilities to function at home, at school, at work, and in social contexts^10,15–17^. Irrespective of the fact that all presentations of ADHD have drastic impacts on day-to-day life, there have been misconceptions that girls/women are unlikely to have ADHD, and so boys are therefore more likely to be referred for assessment, resulting in underdiagnosis for girls^18–21^. Disappointingly, this is still the case despite research as early as the 90’s, and much since, indicating that ADHD is not a gender/sex specific condition, and there are likely more diverse presentations of ADHD beyond the stereotypes^22–24^. In fact, there are concerning statistics on how women are more susceptible to having their diagnosis missed in childhood as, for example, girls are less likely to be referred for diagnosis even when experiencing the same, or greater, level of impairment from ADHD as boys^25^. Additionally, there is a higher ratio of boys to girls in clinical population samples compared to community populations, also indicating that girls are grossly under-referred and therefore underdiagnosed^1,26^.

There are several possible explanations for gender/sex biases in ADHD diagnosis. Despite being separate concepts, terms of sex (biological construct) and gender (social/identity-based construct)^27^ are often used interchangeably by many within society and in psychological literature. This is likely due to minority status of transgender people, and the prominence of cisgender identities in research samples. Both sex and gender are relevant constructs when considering differences in ADHD presentation and diagnosis levels as both societal and biological factors likely play a role^1^. Given (1) the aim of this piece of work is to explore participants experiences from social and emotional perspectives, (2) the current limited understanding of the balance of biological and societal impacts on peoples experience with ADHD, and (3) the cisgender focus of current literature, we will default to gender terms throughout this piece unless specifically referring to biological perspectives (e.g. experiences of puberty or menopause). This is not intended to undermine the difference between sex and gender, but reflects the match between gender being a social/identity-based construct and our research aiming to take a social/identity-based perspective to exploring women’s experience with delayed ADHD diagnoses.

Several intersecting biological, socio-political, and cultural factors could explain why cis-women and girls are more likely to have delayed ADHD diagnoses (for a review see^1^). This can include presentation of symptoms in girls versus boys due to biological differences in likelihood of experiencing certain symptoms, or differences in use of compensatory behaviours to mask symptoms to meet social norms and expectations^19,28–31^. Additionally, even after referral, boys may be more likely to be diagnosed than girls because the criteria outlined in the Diagnostic and Statistical Manual of Mental Disorders^7^ is based on research in which boys are significantly better represented^32^. Thus, the DSM criteria may better represent the typical presentation of ADHD in boys than girls^23,33^.

While explanations for delayed diagnoses of ADHD in girls/women from perspectives of teachers, parents, and medical professionals are important to consider, first-hand perspectives from women with late-diagnosed ADHD are equally important. Holthe and Langvik^30^ highlight that the experiences of women with late-diagnosed ADHD benefit from qualitative research methods, allowing participants to share their perspectives. Their study aimed to understand more about the experiences of women living with ADHD. Holthe and Langvik^30^ interviewed five women (aged 32–50) with late ADHD diagnoses and evaluated their responses using a ‘bottom-up’ reflexive thematic analytical approach^34^. They identified themes which characterised salient views from participants responses including ‘conflict between ADHD symptoms and gender norms and expectations’, which aligns with the suggestion above that masking could contribute to girls’ and women’s late diagnoses. All women recounted noticing early signs indicative of ADHD, and while these struggles were also noticed by parents/teachers, the participants were not referred for diagnosis. Other themes that were uncovered were ‘from unidentified childhood ADHD to adult diagnosis’, ‘present main symptoms and challenges’ ‘stigma of ADHD: ‘people think it’s a fake disease,’ and ‘managing ADHD symptoms and identifying strengths’. Emotional and psychological impacts of participant’s late diagnoses were uncovered in these themes, but this aspect was not central to their aims of understanding how the ADHD diagnosis influences their adult life. Participants, for example, reported that stigma and public misconceptions meant that their difficulties were minimised and dismissed and reported that lack of explanation for their difficulties caused participants to blame themselves, and combined with frequent criticism for their behaviour, this negatively affected their sense of self.

Attoe and Climie^35^ also investigated the impact of undiagnosed ADHD on women. Authors carried out a systematic review on eight papers using reflexive thematic analysis^34^. Similarly to Holthe and Langvik^30^, they identified late-diagnosed ADHD as having negative impacts on psychological wellbeing. Participants also struggled with relationships and self-control but found self-acceptance post-diagnosis. However, as was the case with Holthe and Langvik^30^, seven of the eight reviewed papers used small samples (< 17). In both Attoe and Climie^35^ and Holthe and Langvik^30^ it seems evident that earlier diagnoses would likely have helped participants, but it was unclear what stages of their lives were most impacted, when the earlier diagnosis would have made the most difference, and what aspects of their negative experiences this could have most improved.

Morley and Tyrrel^36^ and Morgan^37^ also demonstrate the benefits of qualitative methods to understand the impact of late ADHD diagnosis on social, academic, and psychological functioning. Both studies used interview methods to understand adult women’s perspectives of living with ADHD^35,36^ and being diagnosed as an adult^36^ . Participants from both papers reported a lack of recognition of ADHD by professionals (instead being labelled as depressed/anxious). They felt dismissed by medical professionals and felt that healthcare accessibility was a barrier to diagnosis. Participants reported long-term mental health issues, with poor self-esteem pre-diagnosis. They masked symptoms to fit social gender-based expectations of behaviour and lack of diagnosis affected their academics, home life, work life, and relationships. These results concur with previous work (e.g.^3–6,10,15–17,19,30,31^), however all eight participants (age range 20–5) in Morley and Tyrrell’s work, and most participants (age range 19–56, almost half under 25) in Morgan’s work were university students. Morley and Tyrrell also excluded participants with co-morbid diagnoses of other serious mental health disorders (e.g. depressive disorder), making results potentially unrepresentative of people with ADHD more broadly given the high comorbidity of ADHD with other mental diagnoses^5,38^ and the possibility that undiagnosed ADHD may be a contributor to development of such conditions^39,40^. Morgan^37^ and Morely and Tyrrel’s^36^ work centred more around the psychological impact of late diagnosis, but again did not examine explicitly how different stages of life were impacted. This is important to know for health services and policy makers to know where to best allocate resources, and the types of resources to allocate, when trying to support people who have been failed by medical systems previously, or prevent such failings going forward.

Despite a dearth of research on ADHD girls/women in comparison to boys/men, the research which has been undertaken indicates that their daily functioning is impaired; including academically, emotionally, and in interpersonal relationships^22,33,41–43^. Concerning statistics include increased risk of substance misuse^5^, domestic abuse and unplanned pregnancy^33^; and self-harm and suicidal behaviour (which is greater than both non-ADHD peers and ADHD boys^44^). These difficulties often persist throughout adulthood^45^, further exacerbated by delayed diagnosis, as comorbidity with other psychological disorders is more likely when women are diagnosed late^39,40^.

Considering the gender bias in diagnosis age of ADHD for women, and the possible severe negative impacts of delayed diagnosis, it is incredibly important to investigate women’s experiences with delayed diagnoses further. Current qualitative perspectives presented about women’s perspectives come from mostly young women in university education, however this is likely not representative of women more widely—especially given the possible impacts of delayed diagnosis on academic achievement, those who may have been most impacted may be excluded from such studies. Additionally, no qualitative work has focused on how participants perceive delayed diagnoses impacted different stages of their life. Thus, the aim of this study was to investigate the psychological and emotional impact of delayed ADHD diagnoses on women of wide age range and with varied academic achievement, specifically exploring how they perceive different parts of their lives to have been impacted by centring their personal experiences of their journey before diagnosis. We posited participants with more diverse day-to-day lives (i.e. not primarily students), and with broader age range, may provide additional perspectives reflecting the lived experiences of women with delayed ADHD diagnoses.

## Methods

### Participants and recruitment

Participants were recruited via Prolific. A Screening study was used to identify eligible participants i.e. UK based, cisgender women, who were diagnosed with ADHD after their 15th birthday (see supplementary materials for details). The minimum age of diagnosis was decided due to 15 being the approximate age when young people in the UK begin to have more freedom and responsibility in key areas of life such as choosing school subjects and sitting exams which may affect access to further education. A delay in diagnosis beyond this age may therefore have a particularly negative impact on quality of life. The Main Study consisted of two parts so as not to fatigue participants completing the survey in one sitting. Given results from the Screening Study, Part 1 of the Main Study was advertised to eligible individuals. Main Study Part 2 was advertised to all participants of Main Study Part 1. 28 participants completed Main Study Part 1; and 26 of those also completed Main Study Part 2.

Participants were aged 19–72 years (*M* = 38.8, *SD* = 13.0) and age of diagnosis ranged 18–62 years (*M* = 35.9, *SD* = 12.7). Mean year of diagnosis was 2020 (range: 2011–2024). All participants were diagnosed with ADHD in the UK either privately or through the NHS. Most participants were white, raised in the UK, and were in employment. 79% had achieved, or were working towards, further or higher education qualifications. Further diagnosis information and sociodemographic details are available in supplementary materials.

### Materials and procedure

Data were collected using an online survey hosted on Qualtrics. The questions were designed to prompt participants to give detailed responses on their personal perspectives and experiences of being late diagnosed ADHD. We used a combination of closed and open answer questions to target participants perspectives on the impact of delayed ADHD diagnoses on participants’ childhood, adolescence, and adulthood, their journey to seeking diagnosis, perceived reasons for late diagnosis, and barriers to diagnosis (see Table 1, and supplementary material). Additionally, 14 Likert-scale questions covered possible impacts of undiagnosed ADHD and factors which contributed to late diagnosis (see Table 2). Participant engagement with the survey material was high: all but one participant provided in-depth answers to the free-write questions. Full survey wording and content, including attention checks, is available in the Open Science Framework (OSF) project linked at the end of this paper.

**Table 1:** Summary of categorical and associated open answer questions from Main Study survey.

Closed-ended question	Open-ended question
Do you think having undiagnosed ADHD had an impact on your childhood experience up to 12 years old?.*	Please use this space to explain your answer to the previous question in detail
Do you think having undiagnosed ADHD into adolescence (12–18 years) had an impact on your experience as a teenager or adult?*	Please use this space to explain your answer to the previous question in detail
Do you think the age that you were diagnosed at had an impact on your adulthood? *	Please use this space to explain your answer to the previous question in detail
When did you first suspect you might have ADHD? Please give your best estimate if you are unsure….**	Please use this space to tell us about the context of your early suspicion, or lack thereof, that you had ADHD
Did anyone around you (e.g., family or friends), tell you that they suspected you had ADHD before you suspected it in yourself?*	Please use this space to explain your answer to the previous question in detail
Do you think societal norms (e.g. social or structural norms) have impacted your experience of life with ADHD?*	Please use this space to explain your answer to the previous question in detail
Did you face structural barriers towards getting diagnosed? (this could include those covered in the previous question or not)*	Please use this space to explain your answer to the previous question in detail
Do you think there is anything that could have made your journey to diagnosis smoother?*	Please use this space to explain your answer to the previous question in detail

*Response options provided were ‘yes’, ‘no’, and ‘don’t know’. ** Response options were by age, point in time/date, or how long ago (measured in year/s and month/s).

**Table 2:** Likert-scale questions (on a five-point scale ranging from strongly agree to strongly disagree) given to participants covering possible impacts of undiagnosed ADHD and factors which contributed to late diagnosis.

As compared to if it were diagnosed earlier, undiagnosed ADHD had a significant impact on my experience…
…as a child …as an adolescent …as an adult
My late ADHD diagnosis has had a significant impact on…
…my sense of self …my career …my relationships with friends …my relationships with family …my romantic relationships …my mental wellbeing
Please rate how much you agree the following factors significantly contributed to your late diagnosis of ADHD, i.e. that you were diagnosed as an older teenager or as an adult and not as a child
My gender Medical system structure Ethnicity Concurrent diagnoses (e.g. anxiety, depression, autism, POTs, hyper flexibility) Socioeconomic factors

#### Positioning statement

This study took a contextualist constructivist approach (CCA) which proposes that knowledge is unavoidably subjective; affected by differing contexts such as the participants’ and researchers’ perspectives and the views of the scientific field^46^. To allow readers to consider the influence of authors perspective on the research we outline our position as follows. Both authors identify as cisgender women and were raised in the UK. EH has British heritage, and HKW is of British and Japanese heritage. EH and HKW were both diagnosed with ADHD as adults. Our position as researchers with shared characteristics with participants allows us to empathise with their experiences and therefore analyse and present findings from a more humanistic perspective^46^. We were also aware that there was a potential influence of our preconceived views upon analysis. To address this, we actively paid attention to minority views within the participant responses, and alternative views and experiences to our own.

#### Qualitative analytical procedure: Template analysis

We used template analysis^47^ to analyse open-ended question responses using NVivo^48^. Template analysis fits well with CCA^47^ as both support the collection of qualitative and subjective data. Template analysis recognises subjective reports as valuable insight into women’s experiences of late-diagnosed ADHD, rather than as affecting reliability and validity of research. Following King’s^47^ process, we used previous research findings^3–6,18–25,28–32,35–37,39–44^ and the present research questions to produce an initial coding template containing key a priori themes expected to be found in the text (see supplementary materials for the initial coding template and further information on the template analysis process). In template analysis, the coding template provides an initial guide for the coding process, but is continuously updated based upon the data. HKW conducted initial qualitative analyses. Codes and themes for analyses were generated through an iterative process through HKW revision, and through discussion with EH. After HKW completed coding, EH independently second coded all responses based on HKW’s theme and code structure. EH and HKW then constructed the final theme and code structure and reviewed all coding to fit^47^.

Any discrepancies in content coding were reviewed between EH and HKW until agreement was reached for final categorisation of those cases. More specific details of how HKW and EH conducted the iterative process is available in the supplementary materials. We critically evaluated the analysis process independently and via discussion throughout the coding process. In line with the CCA perspective that differing participant perspectives do not need to be seen as de-validating of one another^46^, we specifically considered perspectives which may have represented differing viewpoints.

Additionally, in the results, we briefly cover themes which were not directly related to the research questions to provide context to the main results^47^. We then present findings based on the main themes and use a range of quotes from different participants to illustrate them (as recommended by CCA^46^), including quoting minority experiences to minimise over-generalisation.

#### Quantitative analytical procedure: Binomial tests

We wanted to explore whether distribution of Likert question responses to differed to that of chance. Given low frequency counts in some categories, Chi-square tests were not appropriate to explore whether observed proportions of participant responses to Likert-scale questions significantly differed from expected proportions. The five response options were thus reduced to binary categories: ‘slightly agree’ and ‘strongly agree’ categories were collapsed to produce the broader ‘agree’ category; whilst ‘slightly disagree’, ‘strongly disagree’, and ‘neither agree nor disagree’ categories were collapsed to produce the ‘not agree’ category. HKW then performed exact single-sample binomial tests using SPSS software^49^. Given the relatively small sample size, we must be cautious about non-significant results as the low power means we would be at risk of accepting the null hypothesis when the alternative hypothesis is true (i.e. type 2 errors). The assumption of independent observations was met in all cases.

### Ethics

This study was carried out in accordance with General Data Protection Regulation guidelines and University of St Andrews University Teaching and Research Ethics Committee guidelines (approval code: PS17477). Participants provided informed consent ahead of completing the study. Participants were recompensed in line with Prolific guidelines.

## Results

### Quantitative impact of late diagnosed ADHD on life stages and aspects of life

85% and 92% of participants agreed delayed ADHD diagnosis impacted their childhood and adulthood respectively. Binomial tests indicated that this was significantly more participants than expected by chance (see Table 3). All participants agreed delayed ADHD diagnosis impacted their adolescence. Binomial tests indicated that more participants than expected by chance agreed delayed ADHD diagnosis impacted their sense of self (96%), career (81%), mental wellbeing (96%), romantic relationships (77%), and relationships with friends and family (both 81%; see Table 3).

**Table 3:** Binomial test results comparing observed ‘Agree’/’Not agree’ response proportions of the impact of undiagnosed ADHD and sociopolitical factors’ contribution to late ADHD diagnosis.

Question	N	Observed proportions agree (not agree)	*p*-value
Impact of undiagnosed ADHD on life stages			
Childhood	26	0.85 (0.15)	< 0.001
Adolescence*	25	1.00 (0.00)	NA
Adulthood	25	0.92 (0.08)	< 0.001
Impact of late diagnosis on psychological factors			
Sense of self	26	0.96 (0.04)	< 0.001
Career	26	0.81 (0.19)	< 0.001
Relationships with friends	26	0.81 (0.19)	< 0.001
Relationships with family	26	0.81 (0.19)	< 0.001
Romantic relationships	26	0.77 (0.23)	< 0.001
Mental wellbeing	26	0.96 (0.04)	< 0.001
Sociopolitical factors contribution to late diagnosis			
Gender	26	0.69 (0.31)	0.002
Medical system structure	26	0.81 (0.19)	< 0.001
Ethnicity	26	0.04 (0.96)	< 0.001
Concurrent diagnoses	26	0.77 (0.23)	< 0.001
Socioeconomic factors	26	0.50 (0.50)	0.199

Test proportions were 0.4/0.6 for Agree/Not agree respectively.

*No binomial test was conducted for impact of undiagnosed ADHD on adolescence as all participants responded ‘Agree’ to this statement.

### Quantitative perceptions on factors contributing to delayed ADHD diagnoses

Binomial tests indicated that more participants than expected by chance agreed that gender (69%), medical system structure (81%), and concurrent diagnoses (77%) contributed to their late ADHD diagnosis (see Table 3). A binomial test indicated that less participants than expected by chance agreed that their ethnicity contributed to their late ADHD diagnosis (4%). The one person who agreed with this statement was a person of colour. An equal number of participants agreed and did not agree that socioeconomic factors contributed to their late diagnosis.

### Overview of open-ended answer content

Participant responses to all open-answer questions constituted 20,958 words (Part 1 participant mean = 401, *SD* = 231; Part 2 participant mean = 334, *SD* = 212). Throughout open-ended question responses, participants frequently discussed their ADHD symptoms; hyperactive, impulsive, and inattentive symptoms of both an internalised and externalised nature. Some symptoms which are not officially diagnostic symptoms of ADHD but which have been associated with the disorder were reported^50^ (e.g. emotional dysregulation and Rejection Sensitivity Dysphoria). Participants reported that symptoms affected many areas of life including education, work performance, and romantic and non-romantic relationships, and reported masking symptoms to meet social norms and gender-based expectations of behaviour (Number participants [N] = 8). Conforming to these expectations was thought by some participants to have contributed to lack of recognition of thier ADHD (N = 6). Overall poor mental health was also discussed, with frequent references to clinical and non-clinical anxiety (N = 14) and depression (N = 9). Other indicators of poor mental health were reports of frequent burnout (N = 3), exhaustion (N = 5), suicidal ideation (N = 2), and self-harm (N = 1). Prior to ADHD diagnosis, risky behaviour included substance misuse/addiction (N = 8), disordered eating (clinical and non-clinical) (N = 2), poor financial decisions (N = 8), and risky sexual choices (N = 3). Some participants viewed diagnosis/being treated for other emotional disorders (anxiety, depression, Borderline Personality Disorder/Emotionally Unstable Personality Disorder, Obsessive Compulsive Disorder) as misdiagnosis (3 of the 9 who mention emotional disorders). For the remaining 6 participants who didn’t mention misdiagnosis, 3 participants said they thought the condition developed as a result of the negative psychological impact of undiagnosed ADHD. Three participants reported also having a diagnosis of Autism Spectrum Disorder.

Participants mentioned changes in symptoms/difficulty managing symptoms along with hormonal and/or life changes. Four participants shared how ADHD symptoms worsened with changes in hormone levels, including puberty, premenstrual dysphoric disorder (PMDD) and menopause. The impact of symptoms and lack of diagnosis was reported across the lifespan. Some participants specified increased challenges at adolescence and other stages of life (such as growing independence from their parent (N = 1), moving to university (N = 1), and parenthood (N = 1)), due to increased responsibilities which ADHD symptoms affected their ability to manage.

Participants and those around them had misconceptions and a lack of awareness about ADHD, partly attributed to gender-based research bias. This included ADHD as a disorder primarily, or solely, experienced by boys (N = 7), and a lack of knowledge on symptoms other than externalised hyperactivity (N = 5). Some participants also reported that there were negative societal prejudices about ADHD, with one participant reporting being directly discriminated against at work and another participant at college. Combined with long referral waiting times, referral rejections, lack of follow-up support post-diagnosis, and medication shortages, misconceptions and lack of awareness of ADHD were reported as major barriers to diagnosis and access to support. A few participants also reported that gender norms contributed to their suffering as symptoms did not fit with how they were expected to ‘behave as girls’, resulting in criticism and masking. However, participants believed that awareness and understanding of ADHD has increased in recent years. Example quotes representing these perspectives are available in the OSF project. The psychological and emotional impact of late-diagnosed ADHD was discussed by all participants.

### Psychological and emotional impact of late diagnosis

**Fig. 1:**
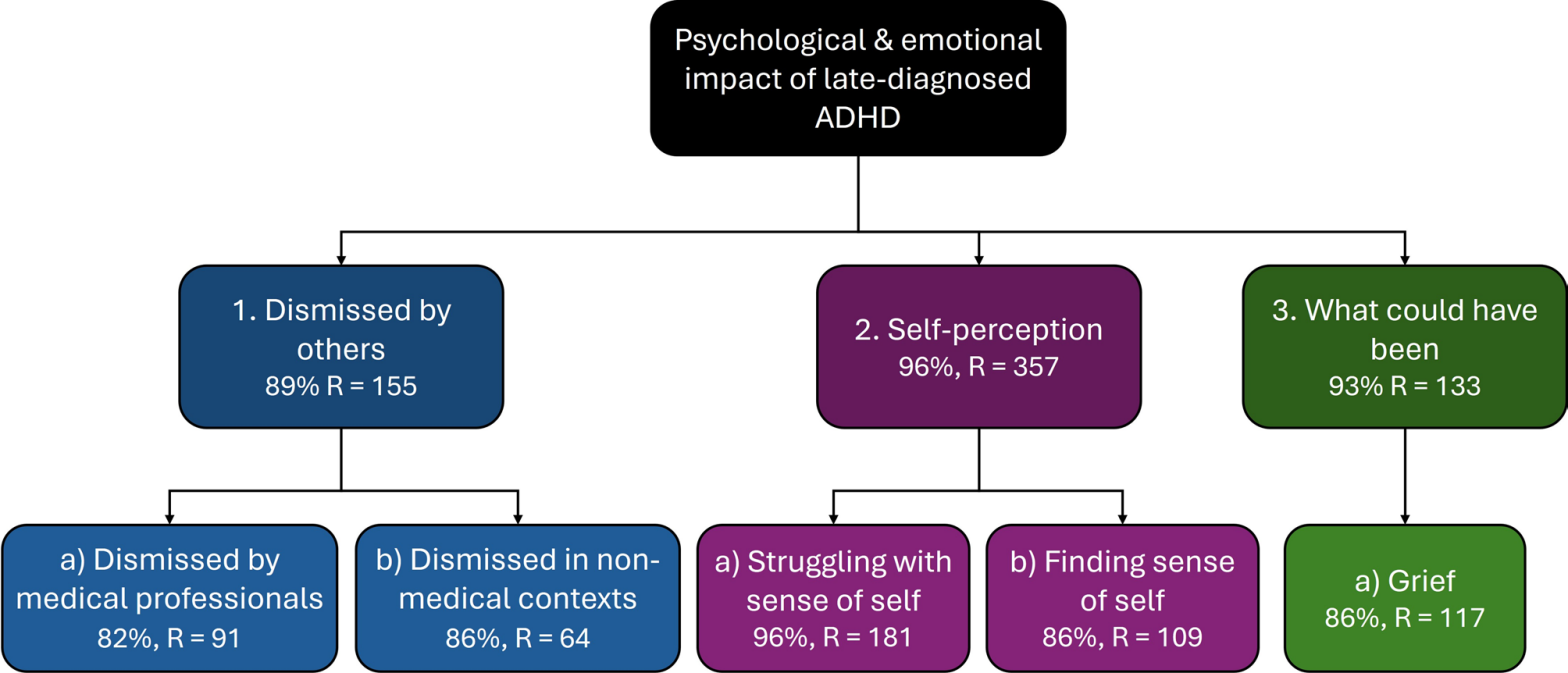
Thematic map including hierarchy of primary themes, and associated subthemes. Where % represents percentage of participants, and where ‘R’ represents number of mentions across all participant responses. Numbers denote themes, letters denote subthemes.

All themes/codes from the a priori template were identified in participant responses to varying degrees. Once the template was restructured to reflect data, a thematic map was produced which illustrates the hierarchy of key themes and subthemes (see Fig. 1). Below we have replaced ages with age ranges, and all names are pseudonyms to ensure anonymity.

#### Theme 1: Dismissed by others

**Fig. 2:**
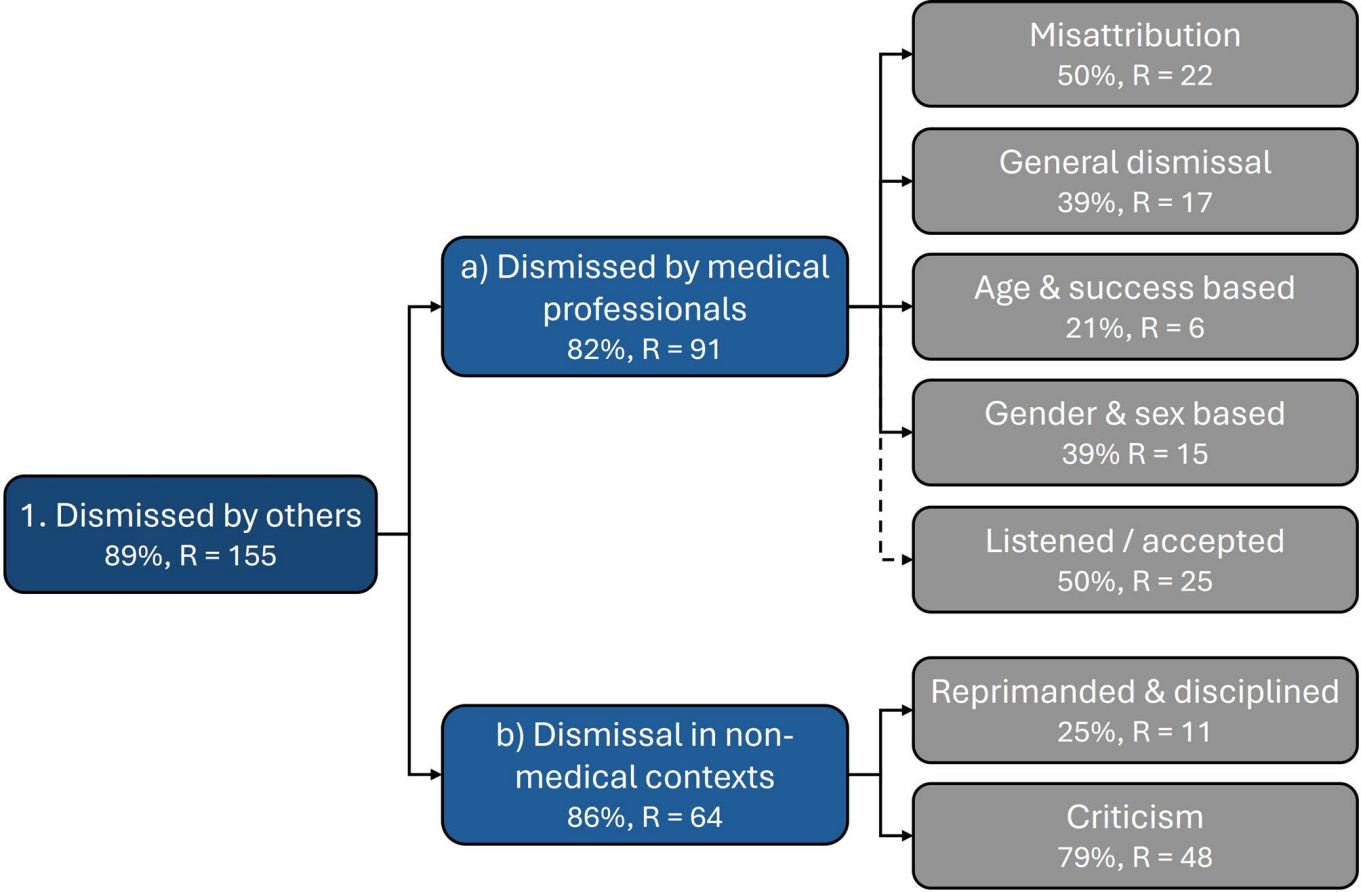
Thematic map of ‘Dismissed by others’ theme, associated subthemes (denoted by letters) and codes (in grey). Where % represents percentage of participants, and where ‘R’ represents number of mentions across all participant responses. Dotted line denotes inversely loaded code.

Theme 1, ‘Dismissed by others’, addresses participants’ reports of being told directly, or through actions, that their struggles were not real, or were dismissed as inherent faults (Number participants [N] = 25 (89%), number References [R] = 155). This includes being dismissed by medical professionals (subtheme 2a; N = 23 (82%), R = 91) and in non-medical contexts (subtheme 2b; N = 24 (86%), R = 64) relating to their ADHD symptoms and associated difficulties (See Fig. 2). Dismissal was observed in participant responses in the context of considering the impact of late diagnosis on childhood, adolescence, and adulthood (see supplementary material).

*Subtheme 1a: Dismissed by Medical Professionals*: 19 (68%) participants reported being dismissed by medical professionals when attempting to access support for ADHD and/or ADHD diagnosis (R = 66). For example:

“[…] [I] also mentioned [ADHD suspicions] to my GP and I got asked (pointedly) "what makes you think that?"[.] I also have chronic pain issues and I have spent so much time being doubted and gaslit in the NHS that I didn’t have the energy to pursue it. I was [later] referred to mental health services […] and after spending a full hour with pages of notes detailing my ADHD symptoms to the nurse, they refused to refer me for a diagnosis. […]” ~ Kirsty (Included in response to question [Q.] 20, age 30–39)

“[…] [O]nce I started noticing my own symptoms of ADHD as symptoms, I tried to seek out a diagnosis for years. However, I had a mental health team who were unwilling to help me and the process of going to the GP about it was very long at the time. I also had experiences of going to the GP for other issues and being dismissed and sent out the door without really being listened to. […]”~ Michelle (Q.25, age 25–29)

The quotes above demonstrate wider participant’s feelings of being gaslit or not taken seriously, and medical professionals (e.g. GPs, nurses, mental health team) seeming unwilling to help (N = 11 (39%), R = 17). They reported feeling dismissed in general, not only regarding their ADHD.

Fourteen (50%) of participants also reported symptoms being misattributed to anxiety, depression, or hormones (R = 22):

“[...] It was put to me strongly, and more than once, by professionals in the NHS that I was simply anxious or depressed, that it wasn’t ADHD. It was [a] huge vindication that my depression and anxiety got much better when I was diagnosed with and medicated for ADHD.” ~ Kirsty (Q.32, age 30–39)

“[…] Professionals were [..] too quick to label me as depressed and anxious instead of investigating and listening to me[.]”~ Jessica (Q.32, age 25–29)

As demonstrated by Kirsty and Jessica, these participants felt their symptoms were not solely attributable to other issues, yet medical professionals did not listen. Participants also reported that they were dismissed by professionals who believed that their difficulties were not significant enough to require a diagnosis if they had lived without a diagnosis or support into adulthood and still coped/been societally successful (N = 6 (21%), R = 6), for example:

“I had a hard time getting my doctor to agree to refer me for an assessment. His attitude was that [I']m ok as [I’]ve got this far through life. My [l]ife has been one drama / chaos / traumatic event after another.” ~ Emma (Q.25, age 50–59)

Dismissal due to their gender and/or sex was also reported by 11 participants (39%, R = 15), for example:

“[...] my gender certainly did [contribute to my late diagnosis as] being a female[,] its harder to get di[a]gnosis plus having other mental health conditions didn’t help as they affected my diagnosis all the [time.]” ~ Georgia (Q.32, age 18–24)

In contrast, four participants (14%) reported only positive experiences with medical professionals (e.g. being listened to/accepted) in the context of seeking an ADHD diagnosis. Ten (32%) participants reported a combination of positive and negative experiences, nine (36) only mention dismissal without mentioning positive experiences. Overall positive experiences were reported R = 25 times (quotes available on OSF).

*Subtheme 1b: Dismissal in non-medical contexts* addresses participant reports (N = 24 (86%), R = 64) of being dismissed by people who are not medical professionals. For example, the women in our study were criticised for presentation of their symptoms as ‘unacceptable’ behaviours and/or their symptoms dismissed as inherent faults in them as individuals (N = 22 (79%), R = 48). For example, ADHD manifestations being dismissed as them being badly behaved:

*“[…]*I was dismissed as naughty and I didn’t understand why my brain wouldn’t let me be like everyone else.[…]” ~ Kate (Q.13, age 25–29)

“I was labelled as a ‘difficult’ child who would not behave or pay attention in class. At the age of eight I found out that people (including my parents) found me very bad-tempered and difficult to manage.[…]” ~ Heather (Q.13, age 50–59)

Some participants also had their symptoms dismissed as lack of effort:

“[…] I feel like if I had [a diagnosis] earlier, I could have got help for it at school instead of just being branded lazy and as someone who never stuck anything out. […]”~ Michelle (Q.13, age 25–29)

“[…] I was just considered bright but talked too much, was disruptive, could try harder, could do better etc[.]”~ Emma (Q.23, age 50-59)

Some participants also reported their ADHD symptoms were dismissed as ‘weird’, and they described a need to conform to avoid negative perceptions:

“Growing up when I did and coming from a family that expected impeccable behaviour felt like a nightmare. I was made to feel like the black sheep of the family, because even though I conformed all I could there were times I couldn’t control my so-called weirdness.[…]” ~ May (Q.21, age 50–59)

Participant’s symptoms were not just criticised, but seven (25%) participants also reported being punished or reprimanded for the manifestation of their ADHD:

“[...] I lacked the ability to stick at anything. Concentrating at school as a teen was difficult, I was often quick to react to certain situations and often in trouble yet deemed very likeable by most teachers.[...] ~ Meg (Q15, age 30–39)

“[...]I would get into trouble for interrupting and for stopping listening when I felt I already knew the gist of what teachers and other adults would say.” Zoe (Q13, age 30–39)

#### Theme 2: Self-perception

**Fig. 3:**
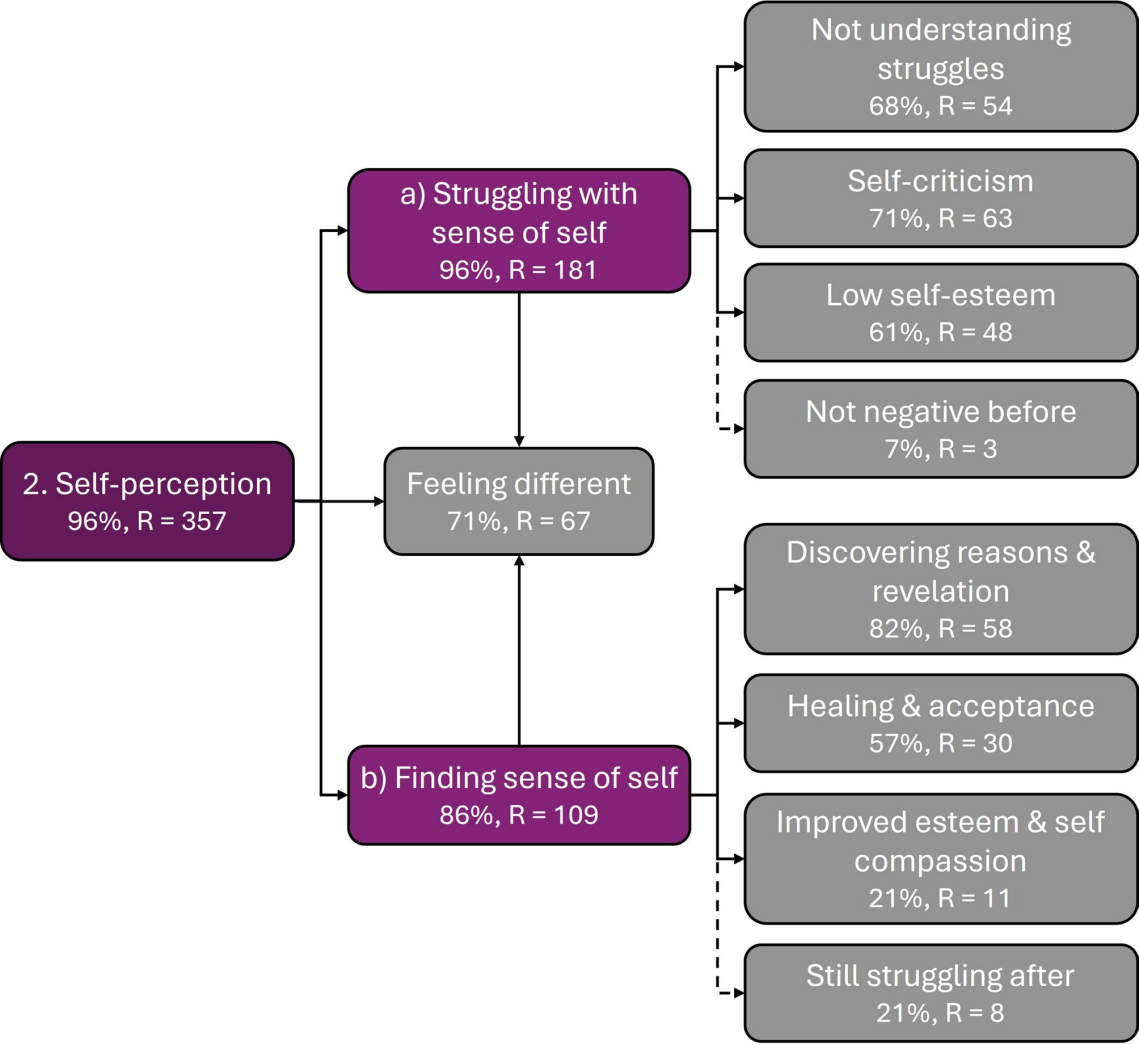
Thematic map of ‘Self-perception’ theme, associated subthemes (denoted by letters) and codes (in grey). Where % represents percentage of participants, and where ‘R’ represents number of mentions across all participant responses. Dotted lines denote inversely loaded codes.

Theme 2, *‘Self-perception’*, addresses participants’ understanding of who they were as a person, their self-esteem, and understanding of their behaviours/experiences (N = 27 (96%), R = 357). Participant responses frequently expressed a transition from struggling with sense of self pre-diagnosis (subtheme 2a), to finding sense of self post-diagnosis (subtheme 2b, see Fig. 3). Note that when using the term ‘diagnosis’ in the context of pre- and post-diagnosis here, we include initial self-diagnosis when participants identified their symptoms as ADHD. Self-perception codes were observed in participant responses in the context of considering the impact of late diagnosis on childhood, adolescence, and adulthood (see supplementary material).

Twenty participants (71%) described feeling different to others (R = 68), with mentions from contexts both before and after diagnosis. Sometimes this was expressed through negative comparisons to others, through descriptions of feeling ‘weird’ or not ‘normal’, describing feeling like something was wrong with them, or describing struggling to be able to do the things other people seemed capable of. For example:

“I compared myself to other people of my same age and wondered why they are able to do the things that I can’t do such as socialising well.[…]” ~ Kim (Q.17, age 18–24)

“I don’t know how to describe it properly but I’ve spent a lot of years thinking I’m not ‘normal’. It felt like everyone was playing game I didn’t get the instructions to.[…]” ~ Alison (Q.21, age 40–49)

*Subtheme 2a: Struggling with Sense of Self*: 27 (96%) participants referred to the negative impact of lack of diagnosis on their sense of self (R = 181). For example, participants expressed not understanding why they struggled (N = 19 (68%), R = 54):

“[…] I feel that when I was a child, there were so many things I wanted to do, but never understood why I would [not] see things through. […] I didn’t know why I would do the things I did. I felt like I never really knew or understood myself. Romantically, I had trouble committing to a relationship, I was always seeking the rush of casual relationships, again, I never knew why this was.” ~ Fiona (Q.30, age 40–49)

Without explanation for their difficulties, participants often reported being self-critical (N = 20 (61%), R = 63) and express low self-esteem due to lack of diagnosis (N = 17 (71%), R = 48):

“[…] I just felt a failure and very negative about myself, like I was no good at anything.[…]” ~ May (Q.16, age 50-59)

“[...] I was constantly doing and saying things I shouldn’t have.” (Imogen, Q.15, age 40–49)

“[…] [H]ad I been diagnosed as a child, I would have had more understanding of what was causing issues as I got older, I could have identified supports or coping mechanisms sooner, and I would have spent less time developing major issues with self[-]confidence and sense of self.[…]” ~ Sophie (Q.30, age 40–49)

“[...] I literally hated myself for most of my life and I think that might have been avoided had I been diagnosed earlier.” ~ Sam (Q.30, age 25–29)

Two participants also described positive or non-problematic aspects to their sense-of-self prior to diagnosis (R = 3), (e.g. that it endowed them with positive attributes such as curiosity and the ability to easily multitask) but both also described negative aspects of sense-of-self prior to diagnosis.

*Subtheme 2b: Finding sense of self*: 24 participants (86%) reported improved sense of self post ADHD diagnosis (R = 109). This included increased understanding of past and present difficulties as a component to their improved sense of self (N = 23 (82%), R = 58):

“[…] Post getting a diagnosis and having that evidence I felt I could drop the act a little and had more of an understanding for why I felt everyday things were harder.” ~ Carla (Q.21, age 25–29)

“Well looking back it all makes sense on why [I] couldn’t sit still and do things normal people would. […]”~ Georgia (Q.17, age 18–24)

Some noted a sense of revelation upon discovering that they had ADHD:

“[…] When I started to read up on it, I felt like I had found my people! So many of the symptoms were the things I had been struggling with my whole life and feeling that I was uniquely defective in being unable to master them. […]”~ Heather (Q.17, age 50–59)

“[…] [My diagnosis] helped me make peace with my past choices and mistakes and now I know why I behaved in certain ways throughout my childhood and adolescence and that has been a real revelation to me.” ~ Joanna (Q.16, age 40–49)

Throughout responses, participants described feelings of healing and acceptance brought about by discovery of their ADHD (N = 16 (57%), R = 30). Six participants (21%) also described increased self-esteem and compassion (R = 11). Some examples of these codes include:

“My life changed when I was diagnosed. […] Once I learned more about [ADHD] I was able to accept myself more and work with my neurotype rather than constantly railing against it and ultimately setting myself up for failure. Now I can give myself strategies and allowances and so can work and [go to] uni[versity], which means I actually have a chan[c]e at success now, and not hating myself anymore.” ~ Kate (Q.16, age 25–29)

“[…] The diagnosis of ADHD was a huge help and really allowed for me to heal.” ~ Zoe (Q.17, age 30–39)

“[…] Having the ‘label’ made it easier to come to terms with the feeling that I was very different to my peers all my life.” ~ Jane (Q.16, age 60+)

Participants still reported feeling different, but viewed this in a more positive light. For example:

“[...]I will say that I am less harsh on myself and have accepted that my brain works differently to other people’s but it isn’t a personal failing.” ~ Sam (Q.16, age 25–29)

Six participants also reported some negative aspects to their sense-of-self post-diagnosis, some referring to adjusting to the new ‘label’, and others referring to the lasting effects of having endured negative sense-of-self for so many years pre-diagnosis which has continued for them post-diagnosis (R = 7). All of these participants also referred to finding sense of self post-diagnosis in other parts of their responses.

“When I was first diagnosed I kind of felt that there was something wrong with me and it definitely changed how I saw myself.” ~ Emily (Q.30, age 25–29)

“[...]At 26 I am now unsure of who I actually am as a person and what was just ADHD.[...]”. ~ Jessica (Q.30, age 25–29).

#### Theme 3: What could have been

**Fig. 4:**
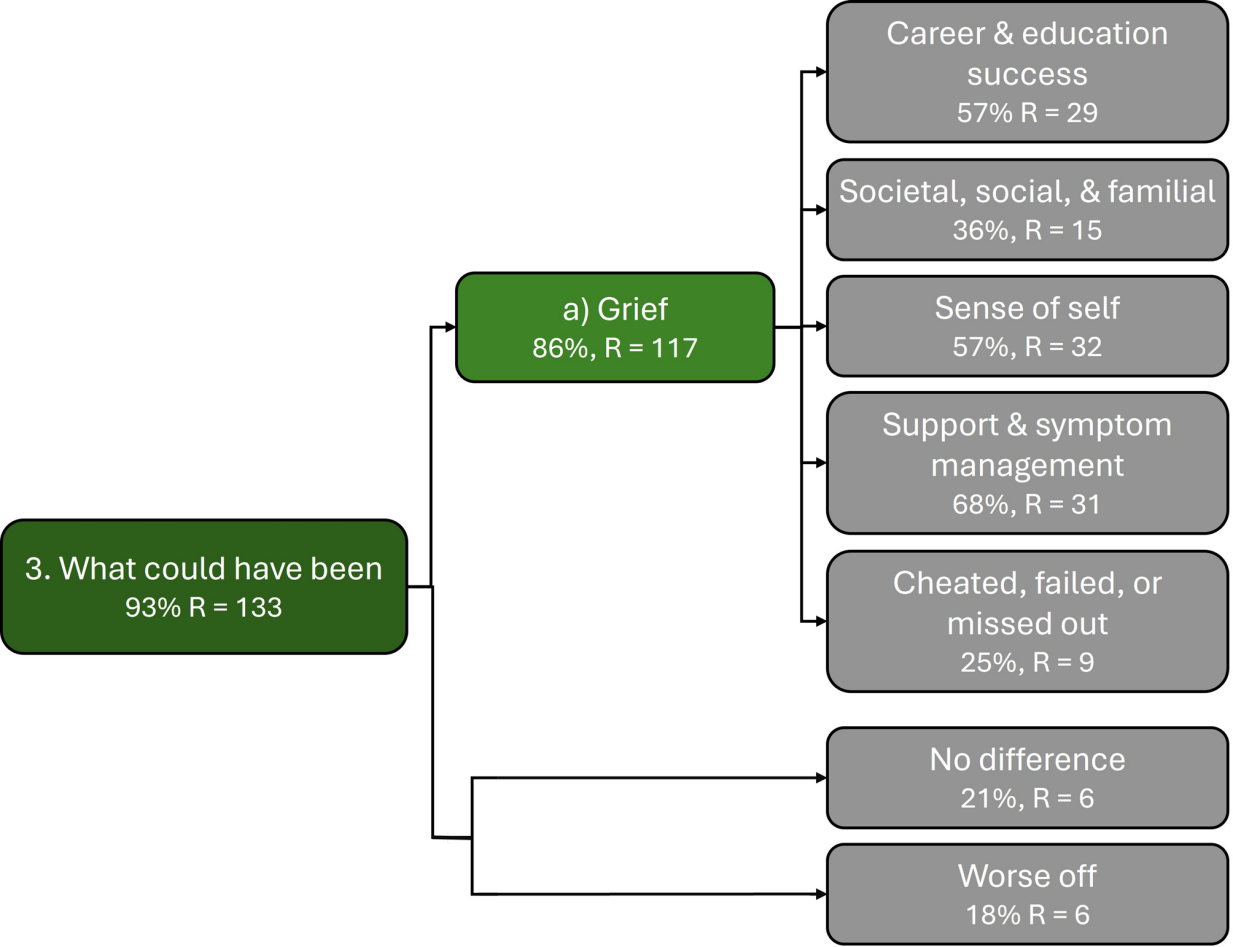
Thematic map of ‘What could have been’ theme, associated subtheme, and codes (in grey). Where % represents percentage of participants, and where ‘R’ represents number of mentions across all participant responses.

Theme 3, *‘What could have been’*, addresses participants reflecting on how their lives could have been if they had a diagnosis earlier (N = 26 (93%), R = 133). Reflection on alternative life experiences was observed in participant responses in the context of considering their childhood, adolescence, and adulthood. There was an overarching sense of grief for the lives they could have led, with 24 participants (86%) reflecting on how life could have been improved with an earlier diagnosis (R = 117) (See Fig. 4).

This included participants indicating that an earlier diagnosis could have enabled them to achieve more professionally and academically (N = 16 (57%), R = 29) and/or in their social or familial lives (N = 10 (36%), R = 15):

“I struggled with my mental health for years, I also found it hard to maintain healthy relationships. I believe had I been diagnosed earlier I would have went on to have a much better career.” ~ Imogen (Q.30, age 40–49)

“[…] [I]f I was diagnosed earlier I think I would be able to be a [better] person and a better partner, not to mention a [better] mother because I would be able to control my thoughts and be more patient.” ~ Jessica (Q.16, age 25–29)

“I think had I been diagnosed when I was younger I would have been more successfully at college; and managed to go to university. [...]”~ Imogen (Q.15, age 40–49)

“I think if I was diagnosed sooner it may have helped me [r]ealise what jobs were not [s]uitable for me and what jobs would [s]uit me best.[...]” ~ Diana (Q.16, age 40–49)

Participants also expressed that earlier diagnosis could have afforded them better sense-of-self (e.g. better self-understanding and mental health) (N = 16 (57%), R = 32), and could have afforded them access to years of support and symptom management that was missed out on (N = 19 (68%), R = 31).

For example:

“[...] Had I been diagnosed earlier, I would have perhaps learned coping mechanisms sooner; had reasonable adjustments at university and work; found a medication that worked sooner; avoided many years of self-doubt; low confidence and thinking I wasn’t good enough. [...]” ~ Sophie (Q.16, age 40–49)

“[...] If I was diagnosed earlier I think I would have enjoyed education a lot more and had appropriate adjustments.” ~ Carla (Q.13, age 25–29)

The emotional impact of reflecting on ‘how their lives could have been’ was salient in responses with participants describing hurt, loss, and feelings of having been let-down with them sometimes explicitly indicating they felt cheated, failed, or that they missed out (N = 7(25%) R = 9). For example, participants mention how the improved self-esteem found post-diagnosis could have been found sooner, and thus prevented years of suffering, for example:

“I feel failed, everything has been such a struggle my entire life and if [I’]d had help sooner, my life may have been a little happier. Now [I’]m just lost[.]” ~ Emma (Q.20, age 50–59)

“I wish I had my life again but with a diagnosis. It makes me so sad to know how better life could have been. It breaks my heart and I struggle to move past that. I feel cheated[.]” ~ Alison (Q.20, age 40–49)

“Everything clicked into place when I was diagnosed at 48. […] I am more content now than I have been my entire life. Occasionally, I get angry that I’d been seeing psychologists, GPs and psychiatrists since I was [5–10 years old] and no one had spotted it before. That’s forty years of living with guilt and shame about who I was, and without access to the information that has since allowed me to understand myself and make the accommodations I need to thrive. It all could have been avoided. I don’t let myself dwell on that, though. Life looks good from hereon in.

One big regret, however, is that I never had children. I love all my friends’ kids […] but I could never see how I could be a mother myself. I was too disorganised, exhausted and emotionally unstable. As I was diagnosed at [45-49 years old], it is now too late. I worry that I’ll be facing a very lonely old age. But again, I don’t allow myself to dwell on that.” ~ Heather (Q.16, age 50–59)

Above, participants expressed feeling let down and grieving for the better lives they could have led with an earlier ADHD diagnosis. Whilst Emma and Alison experienced present difficulties with moving on from feelings of being let down, Heather said that she does not allow herself to ruminate.

Six participants (21%) mentioned aspects of life that they did they did not think a diagnosis would have made a significant difference to their lives (R = 6), and five participants (18%) mentioned areas of life that an earlier diagnosis could have had a negative impact (R = 6). All who mentioned areas where earlier diagnosis wouldn’t have made a significant difference, and three of whom mentioned having a negative impact, also mentioned ways in which diagnosis could have benefited aspects of their lives.

## Discussion

We aimed to investigate psychological and emotional experiences across the lives of women with late-diagnosed ADHD by centring personal experiences and identifying common themes. Participants reported predominantly negative psychological and emotional impacts of ADHD being missed earlier in life, and the societal misconceptions that relate to it. Responses were highly emotionally loaded, and it was overwhelmingly salient across responses that there were significant adverse effects of delayed diagnoses.

Our qualitative analysis exploring psychological and emotional experiences produced three themes. Theme 1, *‘Dismissed by others’*, covered dismissal from medical professionals and dismissal in non-medical contexts (e.g. from parents or teachers). These findings are consistent with previous research which acknowledges misconceptions about ADHD in girls and lack of research which affect public and professional understanding^32^ and recognition of symptoms^25,51^—but crucially, our results testify to such perceptions having concerning impacts on girls with ADHD with them being dismissed for struggles they face daily, in many cases leaving them feeling unseen or gaslit. For example, participants commonly described being criticised and/or disciplined for how their ADHD manifested by family, classmates, and teachers—which ultimately translates to them being dismissed for characteristics that are difficult to help—especially with lack of diagnosis. Furthermore, such dismissal from non-medical and medical personnel was reported at all life stages we asked about, from childhood through adolescence into adulthood.

While there are likely several contributors to underdiagnosis of ADHD in women^7,23,32^, our participant responses show that women with late-diagnosed ADHD themselves perceive their gender to have been a hindering factor in diagnosis which is in line with previous research findings^30,39,52^. Participants also reported medical professionals using characteristics such as their age or success to dismiss the seriousness of their symptoms. Participant’s statement ratings supported this finding when demonstrating that participants believed socio-political factors, including gender, contributed to their late diagnoses. There is little work on ADHD which addresses participants experiencing dismissal based upon their ability to survive without a diagnosis for a major part of their life, but reports from our participants underline those found by Morgan^37^. This highlights an important area for society to reflect upon, especially within the context of medical systems, workplaces, education places where values such as equality, respect, dignity and compassion (particularly within healthcare services) are promoted as values they aim to abide by (e.g. see NHS constitution for England^53^). Participants also covered generally feeling unheard by professionals, and their symptoms being misattributed to other causes. While some research^54^ finds that one reason for delayed diagnosis is that chief complaints are not often about ADHD-like symptoms, our participants often said they were seeking support for their ADHD symptoms, and were dismissed even when they explicitly expressed suspicions of having ADHD. It is also possible that the symptoms presenting in this sample were not those seen as ADHD’s ‘signature’ symptoms, given that ADHD symptomatology appears to vary depending on sex, which may have contributed to their late diagnoses^1^. Additionally, while participants may have been seeking support for comorbid issues, such as anxiety or depression, its possible that these were symptoms in themselves of the undiagnosed ADHD^39,40^—as some participants mention their anxiety and depression improving post diagnosis. This highlights the importance of clinicians exploring potential causes for mental health struggles, and exploring differential diagnosis when they observe symptoms in patients. Future work could also explore this explicitly by, e.g. asking participants timelines of when they approached clinicians for support for ADHD and non-ADHD related symptoms. It is disheartening that lack of understanding from medical professionals led our participants to be dismissed, but our work further stresses the gravity that societal perceptions of function does not negate the severity and need for treatment of internal psychological distress.

Participants were not only criticised by *others* for their symptoms, but this seems to have been internalised and developed into criticism of *themselves*, as reflected within Theme 2, ‘Self-Perception’. This theme covers participants struggling with sense of self predominantly prior to diagnosis, and their recognition of finding sense of self post diagnosis. Participants expressed struggling with lack of understanding for their issues, often having self-critical mindsets and low self-esteem. This underscores and affirms the importance of similar findings from Holthe and Langvik^30^, as we found these difficulties are also salient in a larger and more diverse sample. Despite the difficulties experienced pre-diagnosis, most participants found discovery of their ADHD validating, giving them the opportunity to better understand themselves, and facilitating better self-esteem, self-compassion, healing, and acceptance. While diagnosis was mostly described positively, our quantitative findings further supported the adverse impact of delayed diagnosis on sense of self^30^, with the majority of the women in our study reporting that suffering could have been avoided if they were diagnosed earlier, as demonstrated in Theme 3 ‘What could have been’.

Theme 3, ‘What could have been’, encompassed participants reflections on what their life may have looked like if they had received a diagnosis earlier in life, with emotionally loaded descriptions of, for example, how they could have avoided years of unhealthy coping mechanisms, mental health struggles, and low self-esteem. Participants ratings of Likert-scale statements also illustrated that late-diagnosis considerably negatively impacted perceived wellbeing, relationships (supported by Attoe and Climie^35^), and careers. This demonstrates that it is not just the ADHD symptoms which affect women with ADHD, but also the delay in diagnosis itself.

Considering all themes together, it is clear that diagnosis, or lack of diagnosis, does not just impact one aspect of life. If participants had been able to receive diagnoses earlier in life, not only could they have pursued treatment/support for ADHD symptoms (which may have made some daily processes easier), but they could also have been protected by legislation such as the Equalities Act^55^ when at work or in education, and would have been in legal standing to receive adjustments for their disability within such contexts. With earlier diagnoses, these women may have received more tolerance and understanding from people in society about their manifestations of their ADHD, potentially eliminating years of criticism, discrimination, and development of toxic self-schemas.

In conclusion, while ADHD is a lifelong neurodevelopmental condition, women have been struggling with avoidable additional hardships as a result of their diagnoses having been missed for years of their life in addition to the symptoms themselves. Overall, the experiences of these women with late-diagnosed ADHD starkly demonstrate the lack of support they received from society and medical professionals. Participants were primarily prevented from receiving diagnoses due to misconceptions around ADHD in girls and women, lack of research, and lack of awareness. Instead, their symptoms were dismissed by others, and they struggled with poor sense of self. While our participant sample has a wide age and education background, work with samples who are diverse in other ways (e.g. gender and ethnically diverse) may unveil other amalgams of experience—especially given that intersectionality is known to impact experiences within society.

The primarily qualitative methodology of this study was advantageous due to the explorative nature of the research, providing rich and in-depth information which quantitative methods may not. Our study focused on the experiences of cisgender women with delayed diagnoses of ADHD due to the high rates of underdiagnosis in this population, but it is possible that our findings are not gender-specific, as the delay of ADHD diagnosis could affect the life course of anyone with ADHD irrespective of gender. Future research comparing people with ADHD’s experiences across gender identities and those with early versus late diagnoses would help to more clearly disentangle the general effects of living with ADHD, impact of age of diagnosis, and gender. Further research using larger samples would also allow the statistical investigation of whether the present study’s participants’ perspectives are generalisable to a greater and more demographically diverse population. In addition, it would be valuable to statistically investigate the potential impact of other factors such as age, socioeconomic status, and ethnicity on the degree of challenges which people with late-diagnosed ADHD can face. In follow-up work, it would be important to explicitly gather information on mental health state (e.g. previously experienced mental illness but not presently, recent onset of mental illness when did not have history of mental illness), other mental health diagnoses such as anxiety, depression, and OCD, primarily physical conditions such as PMDD and Postural Orthostatic Tachycardia Syndrome (POTS), and neurodivergent conditions such as Autism Spectrum Disorder (ASD) and Dyspraxia.

Although there were three participants who reported having an ASD diagnosis within open-ended question responses, the actual number of participants who had an ASD diagnosis, or suspected they did, is unknown as we did not directly ask for this information. Considering high ADHD and ASD co-occurrence rates^56–58^, and underdiagnosis/delayed diagnosis rates of ASD also being particularly high in women^59–62^, future studies which explicitly gather this demographic information would allow for differential examination of those with both ADHD and ASD, and those with ADHD alone.

Further, there are striking similarities between our participants’ reported experiences of late-diagnosed ADHD and previous reports from women with late-diagnosed ASD^63–65^.This is perhaps indicative of the similar challenges both populations may face when undiagnosed, such as being dismissed and the lack of understanding of themselves. Consequently, they may develop maladaptive and/or devastating attempts to cope as they self-medicate (e.g. with medicines, alcohol, or illegal drugs) and/or feel they must mask their true selves to fit into society. Such issues with self-concept are addressed in the Conners’ Adult ADHD Rating Scales^66^ (CAARS), which is often administered alongside other diagnostic tools during the diagnostic process. The CAARS addresses “problems of self-concept” as one of the key features of ADHD symptomatology. While some would argue that struggling with self-concept is not a symptom of ADHD per se, but a consequence of not having needs met, exploring these mechanisms further could help our understanding of how to best support those who have suffered through late diagnosis.

Overall, our work demonstrates the wide adversities that delaying diagnosis of ADHD can have, but also the potentially wide benefits earlier diagnosis of ADHD can offer. Given the broad scope of contexts that are impacted by diagnosis, is it evident that change on a public, professional, and structural level is needed. Additionally, this emphasises the importance of continued investment into research on ADHD in women and girls—as with more data to develop our understanding, medical professionals and educators would be better equipped to recognise ADHD symptoms in girls and support their needs. Public awareness campaigns would also allow people like the participants of this study to access information on the varying presentations of ADHD, recognise symptoms in themselves, and seek diagnosis. Additionally, although their diagnosis allowed participants to later discover their sense of self and heal, several participants were still also left grieving the lives they could have led if diagnosed earlier, not only in terms of ‘wishing’ it were different, but also in accepting what can never be. It is therefore imperative that further research on girls and women with ADHD is carried out so that women like the participants of this study can make sense of their experiences earlier, find their sense of self, and avoid unnecessary years of suffering.

## Supplementary Information

Supplementary Information.

## Data Availability

Supporting data for this project is available at https://osf.io/wrv46/?view_only=7112f2a8ff614b6aaa6a177654417ea5. This includes data relating to closed answer responses, example quotes for the themes and codes extracted, summary information, and participant facing documents. In line with the data use agreement to which participants provided consent, full responses are not provided. Additional summary information or example quotes available on request to the corresponding author.
