# Supplementary material for: Adverse experiences of women with undiagnosed ADHD and the invaluable role of diagnosis
Source: Sci Rep. 2025 Jul 1;15:20945. doi: 10.1038/s41598-025-04782-y (PMC12218314; doi:10.1038/s41598-025-04782-y)
Supplement: Supplementary file 1 — Supplementary Information. [file 41598_2025_4782_MOESM1_ESM.docx]

# Supplementary Materials for manuscript *“Adverse experiences of women with undiagnosed ADHD and the invaluable role of diagnosis” by* Holden, E., Kobayashi-Wood, H.

## Supplementary Methods

### Participant recruitment: Prolific screening study

To be able to target participants who meet the criteria of inclusion in our study on Prolific, we first ran a Screening study. The Screening study was advertised to people who had the following characteristics (as reported to Prolific): consider themselves to have ADHD, cisgender women, fluent in English, and UK based. To advertise the Screening study to people with these characteristics, we sued Prolific’s inbuilt pre-screening function. The questions we screened with and appropriate answers to be eligible for to participate in the Screening study were as follows:

1. **ADHD**

Participants were asked the following question: *Do you consider yourself to have attention deficit disorder (ADD)/attention deficit hyperactive disorder (ADHD)?*

- Accepted answers for Screening study: *Yes*

1. **Gender**

Participants were asked the following question: ***What gender are you currently?***

- Accepted answers for Screening study: *Woman (including Trans Female/Trans Woman)*

1. **Cisgender and Transgender**

Participants were asked the following question: ***Does your current gender differ from the one you were assigned at birth?***

- Accepted answers for Screening study: *No*

1. **Fluent languages**

Participants were asked the following question: *Which of the following languages are you fluent in?*

- Accepted answers for Screening study: *English*

1. **UK based**

Location screening wording: Where should your participants be located?

- Accepted answers for Screening study: *UK*

If participants signed up to take part in the Screening study via Prolific, they were then taken to a survey hosted on Qualtrics. The questions included in the Screening study included questions to validate information from Prolific screening criteria, and an additional question asking whether they have a formal diagnosis, and if so, whether they received it before or after their 15^th^ Birthday. See table S1 for full Screening study questionnaire. Screening study participants provided informed consent before being presented with the survey, and were reimbursed £0.30 (estimated completion time 2 minutes).

**Table S1. Screening study questions. Note wording for Prolific screening criteria validation questions take wording from Prolific screening questions**.

| **Question** | **Answer options** |
| --- | --- |
| **What gender are you currently?** | - Closed answer options: - Man (including Trans Male/Trans Man) - Woman (including Trans Female/Trans Woman) - Non Binary (would like to give more detail: _________) - Prefer not to say |
| **Does your current gender differ from the one you were assigned at birth?** | Closed answer options:   - Yes - No - Prefer not to say |
| **Do you consider yourself to have attention deficit disorder (ADD)/attention deficit hyperactivity disorder (ADHD)?** | Closed answer options:   - Yes - No - Prefer not to say |
| **Were you diagnosed with ADHD before or after your 15^th^ birthday?** | Closed answer options:   - I do not have a formal diagnosis - I was diagnosed before my 15^th^ birthday - I was diagnosed after my 15^th^ birthday |
| **What is your Prolific ID number?** | Free-write response space |

Note: Wording for Prolific screening criteria validation questions take wording from Prolific screening questions in line with Prolific exclusion criteria guidelines.

### **Participant recruitment: Main Study**

The Main Study was advertised to the 44 individuals deemed eligible form the Screening study using Prolific IDs provided. The Main Study consisted of two parts: 28 participants completed Part 1; and 26 of those also completed Part 2. Median time between completing Part 1 and Part 2 was four days (range: 4-15). Prolific IDs were used to match participants across study parts and were then deleted and replaced with pseudonyms for storage, analysis, and reference to in this paper.

### **Additional diagnosis information**

Participants were aged 19-72 years (*M* = 38.8, *SD* = 13.0) and age of diagnosis ranged 18-62 years (*M* = 35.9, *SD* = 12.7). Mean year of diagnosis was 2020 (range= 2011-2024). All participants were diagnosed with ADHD in the UK: 42.9% (*N* = 12) via public sector, and 57.1% (*N* = 16) via private sector). 67.9% (*N* = 19) of participants were diagnosed with ADHD combined type, 25.0% (*N* = 7) with inattentive type, 3.6% (*N* = 1) with hyperactive type, and 3.6% (*N* = 1) were unsure. Further sociodemographic information is available in Table S2. In summary, most participants were white, raised in the UK, and were in employment. 79% had achieved, or were working towards, further or higher education qualifications.

***Table S2: Additional participant demographic information***

| **Variable** | **Number participants per category** |
| --- | --- |
| Age* | 3 aged 18-24  7 aged 25-29  6 aged 30-39  6 aged 40-49  4 aged 50-59  2 aged 60+ |
| Age of diagnosis* | 6 aged 18-24  4 aged 25-29  7 aged 30-39  6 aged 40-49  4 aged 50+  1 not stated |
| Country brought up in | 26 in UK (17 England; 4 Scotland; 2 Northern Ireland; 3 in more than one UK state);  1 in USA;  1 in UK/USA |
| Place of diagnosis (private sector; public sector) | 18 in England (10; 8)  6 in Scotland (4; 2)  3 in Northern Ireland (1; 2)  1 in Wales (1; 0) |
| Ethnicity | 25 white (European, British, and/or Scottish)  1 Asian Chinese;  1 white and African Caribbean;  1 ethnic heritage from multiple Asian and European backgrounds |
| Employment | 19 employed (12 full-time; 5 part-time; 2 self-employed);  1 due to start job in next month;  4 unemployed and job seeking;  3 not in paid work (e.g. home maker, retired, disabled);  1 semi-retired |
| Highest education level | 9 secondary school (of which 3 are current further/higher education students);  3 technical college;  8 undergraduate degree or equivalent;  8 postgraduate degree (of which 2 completed PhD or equivalent) |

### **Questionnaire design**

In the survey we included wording to encourage participants to elaborate upon their answers to try to elicit fuller and more in-depth responses (e.g. “Please explain your answer in detail.”). As participants could participate at any time from anywhere in the UK, our questionnaire design approach gave us the benefit of being able to collect detailed qualitative data from a large sample, while also balancing the time investment often exerted by researchers during interviews.

Participants were asked to give category responses of *‘yes’*, *‘no’*, or *‘don’t know’* to eight closed-ended questions which had associated open-ended questions asking participants to elaborate on their responses (see Table 2). Fourteen Likert-scale questions covered the different impacts of undiagnosed ADHD and factors which contributed to late diagnosis (see Table 3). Free-write spaces were available for participants to report any thoughts prompted by the Likert-scale questions, as well as to share any other thoughts at the end of Part 1 and Part 2. Full survey available at OSF link.

Participant engagement with the survey material was high: all but one participant provided in-depth answers to the free-write questions. Additionally, six attention-check questions were included throughout the questionnaires (three in each main study part), to which all 28 participants showed high attention: 25 participants passed all attention check questions (including two participants who only completed Part 1 of the main study), and three participants passed 5 of 6 attention checks. Full survey wording and content, including attention checks, is available at Open Science Framework (OSF) project linked in Supplementary Materials.

### **Detailed qualitative analytical procedure**

We used template analysis[49] to analyse open-ended question responses using NVivo [50]. Template analysis fits well with CCA[49] as both support the collection of qualitative and subjective data. Template analysis recognises subjective reports as valuable insight into women’s experiences of late-diagnosed ADHD, rather than as affecting reliability and validity of research. Following King’s[49] process, we used previous research findings and the present research questions to produce a coding template containing key a priori themes expected to be found in the text (see supplementary materials). HKW conducted initial qualitative analyses, for which codes and themes were generated through an iterative process. The initial coding process undertaken was as follows: firstly, HKW familiarised herself with the data by reading participant responses individually, then all the answers to each question, respectively. HKW repeated this process until she had a good overall understanding of the text. Next, a subset of participant responses were coded, considering how areas of text related to the a priori themes. An initial template was then made and used for the remaining data. In line with King[49], HKW periodically checked how well coding fit with themes and revised the template accordingly.

As is common practice when taking qualitative approaches, towards the end of the analysis process, the coding template was reviewed to be in line with the focus of our research question. Next, HKW reviewed relevant codes and considered how they could be hierarchically coded under broader themes relating to our research aims (recommended by [49]). EH then independently second coded all responses based on HKW’s theme and code structure. EH and HKW constructed the final theme and code structure and reviewed coding to fit. Any discrepancies in content coding were reviewed between EH and HKW until agreement was reached for final categorisation of those cases. We critically evaluated the analysis process independently and via discussion throughout the coding process. In line with the CCA perspective that differing participant perspectives do not need to be seen as de-validating of one another[48], we specifically considered perspectives which may have represented differing viewpoints.

***Table S3. A priori theme template***

| **Category** | **Subcategories** |
| --- | --- |
| **Barriers to diagnosis** | 1. Misconceptions about ADHD   *a. Gender- a ‘boy’s disorder’*  *b. Symptoms- a disorder of hyperactivity*   1. Internalised symptoms 2. Healthcare accessibility 3. Misattributions of symptoms to other disorders 4. Lack of research on girls/women with ADHD 5. Masking 6. Lack of recognition of ADHD from parents/teachers/medical professionals |
| **Socio-political and cultural factors** | 1. Gender-based social expectations of behaviour   *a. Criticism*  *b. Masking*  *c. Impact on wellbeing*  2. Social stigma of ADHD |
| **Biological factors** | 1. ADHD as hereditary 2. Hormonal changes impact on ADHD symptoms   *a. Puberty*  *b. Menopause*  *c. PMDD* |
| **Impact of late diagnosis of ADHD** | 1. Education   *a. Primary/secondary school*   1. *Teacher criticism* 2. *Academics* 3. *Social interactions*   *b. Higher education* |
| **Impact of late diagnosis of ADHD cont.** | 1. Education   *a. Primary/secondary school*   1. *Teacher criticism* 2. *Academics* 3. *Social interactions*   *b. Higher education*   1. Relationships   *a. Parents/family*   1. *Criticism*   *b. Friendships*  *c. Romantic relationships/spouses*  *d. Children*  *e. Co-workers*   1. Wellbeing   *a. Self-esteem*  *b. Anxiety*  *c. Depression*  *d. Comorbid disorders*  *e. Misdiagnosis*   1. Work   *a.* *Work performance*  *b. Relationships with co-workers* |

## Supplementary results

Participant responses to all open-answer questions constituted 21338 words (Part 1 = 11652words, Part 2 = 9686 words). The mean participant open answer responses to Part 1 was 402 words (SD = 231, N = 28) and Part 2 was 334words (SD = 212, N = 26).

***Table S4. Distribution of themes coded across questions which explicitly mention a life stage (i.e. childhood, adolescence, or adulthood). Where %s represent percentages of references made per theme/code across the three life stages. Further details of theme distribution across all questions is available in the Open Science Framework (OSF) project.***

| **Theme**  **Subtheme or code**  (Code in subtheme) | **Q.13**  **Childhood (%)** | **Q.15**  **Adolescence (%)** | **Q.16**  **Adulthood (%)** |
| --- | --- | --- | --- |
| **1) Dismissed by others** | 37 | 43 | 20 |
| **a) Dismissed by medical professionals** | 14 | 29 | 57 |
| *(Of which Listened/accepted)* | 0 | 0 | 14 |
| **b) Dismissed in non-medical contexts** | 41 | 46 | 13 |
| **2) Self perception** | 19 | 39 | 42 |
| Feeling different | 31 | 31 | 38 |
| **a) Struggling with sense of self** | 20 | 47 | 33 |
| *(Of which not negative before)* | 2 | 0 | 0 |
| **b) Finding sense of self** | 3 | 17 | 79 |
| *(Of which Still struggling after)* | 0 | 0 | 3 |
| **3) What could have been** | 31 | 45 | 24 |
| **a) Grief** | 29 | 45 | 26 |
| *No difference* | 43 | 43 | 14 |
| *Worse off* | 50 | 50 | 0 |

## Open Science Framework project content:

Additional materials associated with this project can be found on the Open Science Framework (OSF) here: <https://osf.io/wrv46/?view_only=7112f2a8ff614b6aaa6a177654417ea5>

There you can find content such as:

- Full Qualtrics surveys (including attention checks)
- Participant Information sheets, Consent forms, and Debrief Sheets
- Ethical approval
- Project data files
